# The Combined Clinical Diagnosis of TNF-*α*, TSH, and p185 Protein in Breast Cancer

**DOI:** 10.1155/2022/4885378

**Published:** 2022-06-22

**Authors:** Junjie Jiang, Wei Zhang, Hui Liu, Yunyun Yang, Wei Zhang, Chunxia Zang

**Affiliations:** ^1^Thyroid and Breast Dept 1, Extra-Thyroid and Breast Neoplasms 1, Cangzhou Central Hospital, Cangzhou, China; ^2^Outpatient Comprehensive Treatment Room, Cangzhou Central Hospital, Cangzhou, China

## Abstract

**Objective:**

To study the concentrations of tumor necrosis factor (TNF-*α*), thyroid-stimulating hormone (TSH), and c-erbB-2 oncogene protein product P185 in different pathological stages of breast cancer and to analyze their combined clinical diagnosis of breast cancer significance.

**Methods:**

67 breast cancer patients who were treated in our hospital from January 2018 to September 2020 were set as the breast cancer group and were divided into stages I, II, III, and IV according to clinicopathology. In addition, 55 patients with benign breasts who were admitted to the hospital at the same time were selected as the benign breast group, and 60 healthy people in our hospital during the same period were selected as the healthy group. The differences between serum TNF-*α*, TSH, and p185 protein positive rate in 3 groups and the levels of TNF-*α* and TSH and p185 protein positive rate in patients with different pathological characteristics were compared and analyzed, and the differences between the combined detection and the single detection were analyzed.

**Results:**

Compared with the benign breast group and the healthy group, the serum levels of TNF-*α* (44.61 ± 12.54 versus 29.75 ± 10.19 versus 56.87 ± 15.36 versus 102.37 ± 15.36), TSH (0.98 ± 0.13 versus 0.94 ± 0.17 versus 1.17 ± 0.24 versus 1.22 ± 0.15) and p185 protein positive rate were higher in the I-II and III-IV groups, and the difference was statistically significant (*P* < 0.05). TNF-*α* detection sensitivity was 44.74%, specificity was 62.06%, which was higher than p185 sensitivity of 31.01%, specificity of 49.78%, higher than TSH sensitivity of 27.51%, specificity of 39.77%. At the same time, the sensitivity and specificity of combined detection of TNF-*α*, TSH, and p185 protein were 67.35% and 70.41%, which were significantly higher than the sensitivity and specificity of single detection, and the difference was statistically significant (*P* < 0.05).

**Conclusion:**

TNF-*α*, TSH, and p185 protein are expected to be used as auxiliary basis for diagnosis in the future. But in general, the serum indexes in this study had low sensitivity and specificity for the diagnosis of breast cancer, which limited their diagnostic function in clinical use.

## 1. Introduction

Breast cancer is one of the most common malignant tumors in women [1]. In recent years, breast cancer seriously threatens women's health and affects their quality of life. Therefore, early diagnosis is critical to the treatment of breast cancer [2]. The pathogenesis of breast cancer is complex and closely related to genetic factors, age and endocrine factors [3, 4]. In recent years, with the rapid development of molecular biology, more choices have been provided for the diagnosis of breast cancer. Through in-depth research on the occurrence mechanism and therapeutic effect of breast cancer, it is found that the continuous development of inflammatory factors increases the incidence of tumors. For example, chronic cervicitis may develop into cervical cancer, and chronic hepatitis may develop into liver cancer. Cytokines are central to the communication between cells of the immune system and between immune cells and other types of cells, acting through cell-specific membrane receptors, potentially altering the behavior and properties of cells. TNF-*α* is a biologically active polypeptide secreted by macrophages and is the first cytokine used in tumor therapy [5, 6]. p185 is an oncogene c-erbB-2 gene, also known as neu or HER-2 gene, is a cell-derived protooncogene, and its oncogene and its protein product (P185) are overexpressed and amplified in a variety of tumors. The pathological study on the protein product P185 of the c-erbB-2 oncogene was firstly seen in breast cancer, and its role was also clear. It is generally believed that the positive expression of c-erbB-2 protein product can be used as an independent indicator to judge the prognosis of breast cancer [7]. Endocrine and sex hormones are closely related to the occurrence and development of breast cancer [8]. At present, there are few clinical reports on the combined detection of serum TNF-*α*, TSH, and p185 protein in breast cancer. Therefore, this study investigated the effect of combined detection of serum TNF-*α*, TSH, and p185 protein expression levels in breast cancer patients on the diagnostic sensitivity and specificity, in order to provide relevant theoretical basis for clinical diagnosis of breast cancer. In order to understand the changes of TNF-*α*, TSH, and p185 proteins in breast cancer patients, the serum of breast cancer patients, benign breast patients, and healthy women were collected, and the contents of TNF-*α*, TSH, and p185 protein were measured, respectively, and a comparative analysis was made. The report is as follows:

## 2. Materials and Methods

### 2.1. Research Objects

A total of 67 breast cancer patients who were confirmed by surgery and pathology in our hospital from January 2018 to September 2020 were selected as the breast cancer group, including 55 cases of invasive ductal carcinoma, 5 cases of intraductal carcinoma, 3 cases of lobular carcinoma, and 4 cases of mucinous adenocarcinoma. The age ranged from 29 to 72 years old, with an average age of 46.3 ± 7.2 years old. All pathological diagnoses and histological grades were confirmed by two or more qualified pathologists. According to the WHO TNM staging criteria, 16 cases of carcinoma in situ were <2 cm in diameter, 32 cases were tumor size from 2 cm to 5 cm in diameter, and 19 cases were >5 cm. TNM staging is as follows: 13 cases of stage I, 35 cases of stage II, 14 cases of stage III, and 5 cases of stage IV. In addition, 55 patients with benign breast who were admitted to our hospital during the same period were selected as the benign breast group, aged from 28 to 74 years old, with an average age of 47.0 ± 7.1 years old, including 28 cases of mammary hyperplasia, 22 cases of mammary fibroma, and 5 cases of others. There was no significant difference in age, height, and body weight between the two groups (all *P* > 0.05), and they were comparable. In addition, 60 healthy people in our hospital at the same period were selected as the control group, aged from 29 to 70 years old, with an average of 46.8 ± 6.8 years old. There was no significant difference in the general data of the three groups of patients, and there was no statistical significance for comparability (*P* > 0.05). All patients were informed of the experiment and signed the informed consent. This study was approved and supported by the Ethics Committee of our hospital.

### 2.2. Inclusion and Exclusion Criteria

Breast cancer inclusion criteria are as follows: (1) the subject was diagnosed with breast cancer by biopsy pathology or intraoperative pathological examination and met the diagnostic criteria for breast cancer; (2) the subjects did not receive surgery or drug treatment after the diagnosis of breast cancer; and (3) the subject's medical records are complete. Exclusion criteria are as follows: (1) subjects with other malignant tumors; (2) subjects with severe neurological diseases; (3) with immunodeficiency; and (4) patients with significant cardiac, hepatic, and renal dysfunction. Inclusion criteria for benign breast disease are as follows: (1) the subject has been diagnosed by histopathological examination; (2) the subject has no history of malignant tumor; (3) the subject has not received any treatment before blood drawing; and (4) subjects had complete medical records. Exclusion criteria were consistent with breast cancer.

### 2.3. Methods

After hospitalization, 5 ml of peripheral venous blood was drawn on an empty stomach in the early morning from all patients, placed in a sterile test tube, and then centrifuged at 4°C and 3000 r/min for 5-10 min. The supernatant was taken and transferred to a clean sterile tube and then placed in a -80°C low-temperature refrigerator to be frozen for testing. The serum levels of TNF-*α*, IL-6, and IL-8 were determined by enzyme-linked immunosorbent assay (ELlSA). The instruments and reagents were all produced by Siemens, Germany, and all operations were carried out in strict accordance with the instructions attached to the kit. The serum p185 factor level was detected by enzyme-linked immunosorbent assay, and the Metrap185 kit was provided by the Quidel Corporation of the United States. Take a 96-well microplate and wash it twice with washing solution; add sp185 standard substance, high-value solution, low-value solution, serum to be tested, and analysis buffer blank control with different concentration gradients according to the instructions, all of which are 100 *μ*l; add 50 *μ*l of HRP-Conjugate diluent to each well, seal the plate, and place it at room temperature for 2 h in the dark; wash the plate 3 times, once for 30S, and spin dry; Add 50 l each of TMB substrate solution in turn, wait for color development at room temperature and dark for 15 min; add 100txl stop solution, measure the OD value at 450 nm with a microplate reader immediately after mixing, and the serum p185 factor level (ng·ml) was obtained by drawing a standard curve. TSH was detected by enzyme-linked immunosorbent assay (ELISA), using enzyme as a marker, using noncompetitive reaction mode, and developing color by enzyme-catalyzed substrate.

### 2.4. Evaluation Index and Judgment Criteria

The serum levels of TNF-*α* and TSH and the positive rate of p185 protein were compared among the three groups. TNF − *α* > 30 mIU/L was a positive result, TSH > 1.0 mIU/L was a positive result, and p185 protein antibody positive was regarded as a positive result; the ROC curve was used to compare and observe the diagnostic efficacy of the individual detection and the combined detection of TNF-*α* and TSH levels and the positive rate of p185 protein. The combined diagnosis is based on the tandem diagnosis of TNF-*α* and TSH levels and the positive rate of p185 protein. The calculation indicators include the following: sensitivity = number of true positive cases/(number of true positive cases + number of false negative cases) × 100%, specificity = number of true negative cases/(number of false positive cases + number of true negative cases) × 100%, positive predictive value = number of true positive cases/(number of true positive cases + number of false positive cases) × 100%, negative predictive value = number of true negative cases/(number of false negative cases + number of true negative cases) × 100%, accuracy = (the number of true positive cases + the number of true negative cases/(the number of true positive cases + the number of true negative cases + the number of false negative cases + the number of false positive cases × 100%.

### 2.5. Statistical Methods

The SPSS 22.0 statistical software was used for data analysis, measurement data were expressed as mean ± standard deviation (*x* ± *s*), one-way ANOVA was used for comparison among three groups, and *t* test was used for comparison between two groups; enumeration data were expressed by the number of cases or percentages, and the comparison between groups was performed using the *x*^2^ test. *P* < 0.05 was considered to be statistically significant.

## 3. Results

### 3.1. Comparison of General Data of Three Groups of Patients

There was no significant difference in age and number of smoking and drinking among the three groups of patients and no statistical significance and comparability (*P* > 0.05), as shown in Table 1.

### 3.2. Comparison of TNF-*α* and TSH Levels and Positive Rate of p185 Protein among the Three Groups of Patients

The levels of TNF-*α* and TSH and the positive rate of p185 protein in the three groups of patients were compared, the levels of TNF-*α* and TSH and the positive rate of P185 protein in stage III-IV in the breast cancer group were higher than those in the benign breast group and the healthy group, with statistical significance (*P* < 0.05), as shown in Table 2.

### 3.3. Comparison of TNF-*α*, TSH, and p185 Protein Detection Alone and Combined Detection in the Diagnosis of Breast Cancer

**Figure 1:**
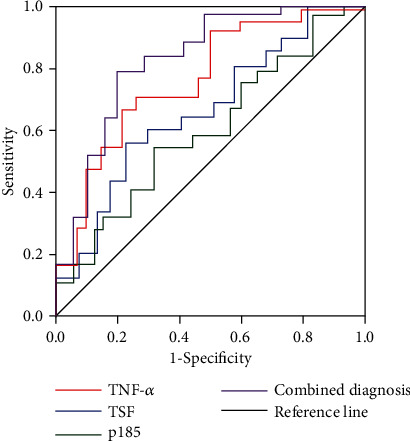
Analysis of the ROC curve of breast cancer in three groups of individual detection and combined detection.

By comparing the diagnostic efficacy of serum TNF-*α* and TSH levels and the positive rate of p185 protein in breast cancer patients, it was found that serum TNF-*α* was the best, followed by p185 and TSH, and the subsided area of the ROC curve of the patients with the combined detection was larger than that of the single detection (the difference was statistically significant (*P* < 0.05)), as shown in Table 3 and Figure 1.

## 4. Discussion

The incidence of breast cancer ranks first among female malignant tumors, and it is a global health concern [9, 10]. The early clinical symptoms of breast cancer patients are not significant, and early clinical diagnosis and treatment is a difficult and hot spot in the field of female cancer research. The selection of appropriate diagnostic methods and breast cancer screening for the high-risk groups is of great significance for the treatment of breast cancer [11, 12]. The current diagnostic methods of breast cancer include ultrasound and pathological biopsy, but the diagnostic value of early breast cancer is relatively limited. With the continuous deepening of serological diagnosis research, new research methods have been brought to the diagnosis of breast cancer. The detection of seromatous tumor markers has received clinical attention because of its simplicity, economy, and high sensitivity and specificity for specific tumors. It has great application value in tumor screening, initial diagnosis, prognosis, efficacy evaluation, and follow-up after treatment. In recent years, there are more and more detection items of tumor markers, but the single detection has the disadvantage of low sensitivity, and the combined detection can improve the sensitivity of diagnosis. At present, the combination of tumor markers is commonly used in clinical detection of breast cancer, which has significant significance in clinical diagnosis. However, there are few studies on the combination of TNF-*α*, TSH, and p185 protein in the diagnosis of breast cancer. This paper mainly discussed its clinical significance in the early diagnosis of breast cancer.

TNF-*α* is an important mediator produced by monocyte macrophages involved in various physiological and immune processes [13]. Studies have shown that in addition to cancerous cells in tumor tissue, 80% of cells belong to stromal cells and inflammatory cells. These stromal cells play an important role in the development of tumor cells, and cytokines produced by stromal cells also form a complex network of tumor regulation [14, 15]. The results of this study showed that the level of serum inflammatory factors (TNF-*α*) in the breast group patients was significantly higher than that in the benign breast group and the healthy group, and with the aggravation of the disease, the serum TNF-*α* level gradually increased. According to the analysis of the reasons, the abnormal immune function of breast cancer patients leads to the hyperfunction of B cells in the body, which promotes the secretion of inflammatory factors such as TNF-*α*, which increases the inflammatory response and causes systemic inflammatory response. In addition, studies have found that as the symptoms of breast cancer patients improve, serum TNF-*α* levels also decrease [16, 17]. Therefore, serum TNF-*α* levels are of great significance for evaluating the progression, diagnosis, and treatment of breast cancer.

Studies have shown that the incidence of breast cancer in people with abnormal thyroid function is significantly increased. The human breast and thyroid belong to hormone-responsive organoids, and changes in endocrine function will lead to corresponding changes in the relevant organs of patients. In tumor patients, thyroid hormone has a certain indicative effect on the metabolic level of the patient's local lesion tissue. By detecting the level of TSH in patients, it can reflect the disease activity of tumor patients to a certain extent, and has a certain monitoring effect on the proliferation process of tumor cells. The results of this study showed that the TSH level in the breast tumor group was higher than that in the benign breast group and the control group, and the TSH level also increased with the increase of tumor stage, which was consistent with the results of Zhang [18] and other studies. The reason for the analysis is that estrogen stimulates the proliferation of thyroid cells and affects both the function of thyroid secretion and TSH levels.

p185 is a transmembrane receptor protein with tyrosine kinase activity encoded by the oncogene C-erbB-2 (also known as neu or HER-2), which can regulate cell proliferation and differentiation after activation [19]. The pathological study of the c-erbB-2 oncogene protein product P185 was firstly found in breast cancer. Some studies believe that in the process of treatment of breast cancer patients, with the prognosis of breast cancer patients, the expression of p185 protein shows a certain correlation. The overexpression of p185 protein level indicates that the risk of recurrence of patients is significantly increased. Current clinical studies have confirmed that P185 combined with CA153 has a high value in the diagnosis of breast cancer. In this study, compared with the benign breast group and the healthy group, the positive rate of serum p185 protein in breast cancer patients was significantly higher, and with the progression of the pathological stage of the patients, it showed a significant upward trend. Lou [20] analyzed the positive rate of p185 protein in breast cancer patients and found that the positive rate of serum p185 protein in patients was significantly correlated with tumor stage, and this study is highly consistent with it.

The results of this study show that TNF-*α*, TSH, and p185 protein are expected to be used as auxiliary basis for diagnosis in the future, but in general, the serum indexes in this study have low sensitivity and specificity for the diagnosis of breast cancer, which limits its diagnostic function in clinical use. Due to the small sample size and single-center data of this study, dynamic monitoring of serum indicators was not carried out, which limited the practical value of the results of this study. However, this study paved the way for future large-sample prospective study and provided a scientific basis for clinical diagnosis.

## Figures and Tables

**Table 1:** Comparison of general data of three groups of patients.

Group	Breast cancer group	Benign breast group	Healthy group	X^2^/F	P
Age				1.817	0.176
Range	29~72 years old	28~74 years old	29~70 years old		
Average age	46.31 ± 7.24	47.03 ± 7.17	46.82 ± 6.89		
Smoking	15	13	10	1.296	0.526
Drinking	14	11	10	1.377	0.503
Tumor size diameter					
<2cm	16	—	—	—	—
2cm ~5cm	32	—	—	—	—
>5cm	19	—	—	—	—
TNM staging		—	—	—	—
Stage I/stage II/stage III/stage IV	13/35/14/5	—	—	—	—

**Table 2:** Comparison of TNF-*α*, TSH levels, and p185 protein positive rate among three groups of patients [n (%)].

Group	n	TNF-*α* (pg/ml)	TSH (mIU/L)	p185 protein positive rate [n (%)]
Stage I-II	48	56.87 ± 15.36	1.17 ± 0.24	28(41.94%)
Stage III-IV	19	102.37 ± 15.36	1.22 ± 0.15	37(55.11%)
Benign breast group	55	44.61 ± 12.54	0.98 ± 0.13	1(1.95%)
Healthy group	60	29.75 ± 10.19	0.94 ± 0.17	1(1.67%)
X^2^/F		20.62	0.93	6.69
P		<0.05	<0.05	<0.05

**Table 3:** Comparison of TNF-*α*, TSH, p185 protein detection alone, and combined detection in the diagnosis of breast cancer.

Diagnosis method	True positive (case)	False positive (case)	True negative (case)	False negative (case)	Accuracy (%)	Sensitivity (%)	Specificity (%)	Positive predictive rate (%)	Negative predictive rate (%)	Standard error	AUC	P
TNF-*α*	17	11	18	21	52.24	44.74	62.06	46.13	72.61	0.042	0.774	0.001
TSH	11	15	17	24	41.79	27.51	39.77	46.92	22.13	0.038	0.690	0.017
p185	12	13	13	29	37.31	31.01	49.78	47.48	31.67	0.031	0.631	0.001
Combined detection	28	18	9	13	55.22	67.35	27.41	56.74	38.89	0.028	0.805	0.017

## Data Availability

The datasets used during the present study are available from the corresponding author upon reasonable request.
